# Hospital and Institutional News

**Published:** 1920-04-10

**Authors:** 


### HOSPITAL AND INSTITUTIONAL NEWS.

### THE WONDERFUL RECORD OF THE WELSH HOSPITAL.

The final meeting of subscribers to the Welsh Hospital at Netley lately took place at the City Hall, Cardiff, when Sir W. J. Thomas presented the final report of the hospital. During the four and a half years of its activities every county in Wales had supported at least one bed, Glamorgan coming first with sixty-three and Monmouth next with twenty-seven. So regular was support given by private persons, sports associations, trades unions and other organisations that only three special appeals were necessary, and there was never a penny overdraft at the bank. The balance sheet showed the total subscriptions to be £80,553, whilst from other sources £7,176 9s. 5d. had been received; this sum included the sale of the building and equipment at Netley. The total expenditure was £78,040, leaving a balance of £9,581 0s. 8d. in the bank and War Loan. It was proposed to use this sum for the foundation of eighteen medical scholarships of £25 per annum at the Welsh National School of Medicine. These would be open to any person who had been for seven years prior to the date of election resident in Wales, including Monmouthshire, or the child or grandchild of a person born of parents resident in Wales, including Monmouthshire, at the time of such person's birth. A gold medal will also be offered every year for the best student in the school, irrespective of nationality.

### ANOTHER PROSPEROUS HOSPITAL.

At the annual meeting of the Savernake Hospital at Marlborough the Rev. A. Joyce Watson, hon. secretary, reported that though the year began with a debt of £361, this had been converted into a credit balance of £138. Commencing in September, patients who were in a position to do so were asked to contribute towards their maintenance, and the request met with a willing response, £88 being added to the income by this means in three months. The Committee had also agreed to take a certain number of discharged disabled soldiers, for whom the Ministry of Pensions paid £188. Mr. H. R. Giffard, who has acted as hon. secretary of the hospital for sixteen years, has resigned. His work has been much appreciated, and its close has been a matter of regret. The Marquess of Ailesbury remarked that it was the most satisfactory report for many years. He congratulated the Committee on the courage with which it had faced and transformed a critical situation. Mr. Herbert Leaf, chairman of the Committee, said that a plan of campaign had been formulated, and the objective in view was an annual income of £3,000. Dr. T. H. Haydon referred to a recent statement that the voluntary hospital system had "broken down," and claimed that the success of the Savernake Hospital proved that the statement did not apply to that part of the country.

### REOPENING OF QUEEN ALEXANDRA SANATORIUM, DAVOS.

All visitors to Davos Platz, in the high Alps, will remember the large white building on the mountain above the little town known to everyone as the Queen Alexandra Sanatorium for British Consumptives. Eighty patients used to be treated there for two guineas a week each. Unfortunately it had to be closed in October 1914, because the war interrupted transit, and in effect cut it off from the Mother Country. The council, not to mention the Swiss, now wish to reopen the institution; and, indeed, it would not look well if this well-known British sanatorium failed to re-start through lack of British support. The difficulties are not only the increased prices, which it is believed would make the cost of maintenance at least five guineas a week, but also the exchange; though this last difficulty needs further explanation. To reopen and re-stock the sanatorium for next winter a sum of £7,000 is required now, and an appeal is made for funds to enable the fees to be reduced as nearly as possible to their original figure. The sanatorium receives patients who could not possibly have the benefit of the Davos climate and treatment without its aid. It is, we believe, the biggest British sanatorium upon the Continent. It should be reopened, and supported by all who know its worth and value British prestige abroad. Mr. E. C. Simmonds, of 18 Finch Lane, Cornhill, E.C. 3, will receive gifts and supply information.

### THE AMERICAN HOSPITAL.

We understand that the work in connection with the founding of an American Hospital in London is proceeding apace under the control of the Governing Board formed for the purpose, and that ex-President William H. Taft has accepted the honorary Presidency in America of this hospital. The honorary President in England is the Earl Reading.

### THE UNMARRIED MOTHERS OF MONMOUTH.

Thanks to a committee of public-spirited women, with Lady Mather-Jackson at their head, Nantyderry House, Abergavenny, will shortly be opened as a maternity home and hostel for unmarried mothers of Monmouthshire. The maintenance charge has been fixed at 15s. weekly, which, it is anticipated, with the promised grant from the Ministry of Health and local support, will be sufficient to balance the accounts.

### ANOTHER HOSPITAL FOR SOUTH WALES.

New hospitals threaten to abound in the thickly populated mining valleys of South Wales. The latest scheme emanates from a committee of the organised workmen, mainly miners, of the Garw and Ogmore Valleys and the Tondu and Kenfig Hill districts, who number some 12,000 in all. The plan is to build on the Abergarw Hill, Brynmenyn, a spot almost in the centre of the area, a well-equipped general hospital. There is little doubt that the miners will ballot in favour of supporting the project by their weekly pence, and it is hoped to enlist the effective sympathy of the employers and the public.

TWO HOSPITAL SUNDAYS.

At the recent annual meeting of the Glasgow Hospital Sunday Fund various methods were discussed for aiding the Glasgow hospitals, which, as we all know, have heavy debts. The most original suggestion was that made by Deacon-Convener Davidson, who proposed that a second annual collection day should be held in the spring, in addition to the traditional Hospital Sunday collection every autumn. The proposal was seconded and carried. There is, however, this difficulty. The success of Hospital Sunday has been so great that many other organisations have laid their claims upon the clergy, who sometimes protest, and not unfairly, that they were not ordained to be professional beggars, and that if the offertory of Sunday after Sunday is devoted to purposes outside Church expenses then parochial needs cannot be met. In moments of impatience the clergy sometimes exclaim against all these collections, unless they remember that Hospital Sunday was their own creation, and was started about seventy years ago by the late Canon Miller, of Birmingham. Consequently, the value of the new suggestion for the annual holding of two Hospital Sundays cannot be judged until the clergy have given their views. What is their opinion of Mr. Davidson's plan?

HOSPITALS AND LAND GIRLS.

The House Committee of Dollis Hill Hospital is anxious to engage a land girl to live, but not to sleep, at the institution. Her duties will be to keep chickens, for fifty of which houses and a run already exist. The land girl will also relieve the odd man of his jobs and help in the garden. The salary is to be 25s. a week, and a profit is expected in view of the price of eggs at the present time.

A PHYSICIAN'S REMARKABLE WILL.

The will of Dr. Ernest Wyndham Cottle, of Yarmouth, Isle of Wight, formerly surgeon to the Scots Guards, and later a well-known skin specialist, is a lengthy document and contains details of unusual interest. The will was recently the subject of an action, and the President of the Probate Court pronounced for its force and validity. It provides first of all that within three days of his death an anatomical examination of his body shall be made by a competent surgeon to ensure certainty of death. £12,000 is left to the Royal Isle of Wight County Hospital and Milligan Convalescent Home, Ryde (as to £4,000 or £4,500 for a "Wyndham Cottle" wing for male patients, and the balance for the endowment of that wing). There are a number of other bequests of money and works of art to relatives, friends, and charitable and other institutions; but the outstanding feature of the will is in respect of the number and extent of gifts to societies for the protection of animals. Dr. Cottle's house and adjoining estate, with £15,000, is allocated to a "Wyndham Cottle Home of Rest for Animals," to be managed, if possible, by the R.S.P.C.A., the Committee of the Isle of Wight County Hospital, Our Dumb Friends' League, and, if in existence, by the Isle of Wight Society for the Prevention of Cruelty to Animals. £2,000 is left to the National Canine Defence League, £1,000 to the Metropolitan Drinking Fountain and Cattle Trough Association for erecting cattle troughs for all animals, and part of the residue to Our Dumb Friends' League and the Royal Society for the Prevention of Cruelty to Animals. The benefits to the last-named Society are to accumulate for twenty-one years, or until certain gentlemen, mentioned by name, have ceased to be connected with the Society. Dr. Cottle was apparently opposed to experiments on animals, for he left £2,000 to the National Anti-Vivisection Society, and directed, in respect of the new Home of Rest for Animals, that no medical, surgical, or scientific research or investigation is to be carried on. If Dr. Cottle's body is not buried at Southampton all money bequests are to be void and the estate revert to the Chancellor of the Exchequer for the reduction of the National Debt. The Chancellor is similarly named as contingent reversionary legatee on the failure of the other trusts or in the event of the will being set aside as regards the bequests for animals. Dr. Cottle evidently had definite knowledge of what he wanted, and took infinite care that his wishes should be carried out.

DR. ADDISON AND MATERNITY SCHEMES.

Speaking at a recent meeting of the Birmingham and Midland Hospital for Women and the Birmingham Maternity Hospital, Mr. W. A. Cadbury, the Lord Mayor, made an interesting announcement. For some months, he said, the Public Health Committee of the Corporation had been drafting a maternity scheme which would give special accommodation to every case in properly equipped centres, with adequate medical attention and nursing by midwives who were fully-qualified nurses. This scheme was submitted to the Ministry of Health, but only partly approved, because the Ministry confessed to a scheme of its own which it did not want to prejudice by any grant of special facilities to Birmingham.

AN ACTUARIAL FALLACY.

Major L. W. Matthews, the Hon. Treasurer of St. Mary's Hospital for Women and Children, Plaistow, suggests that as the net deficiency of Metropolitan Hospitals at the end of 1919 was £210,000, and the King's Hospital Fund distributed a somewhat similar sum, as a temporary measure the difficulty might be solved by the Government making a grant to the King's Fund of pound per pound for distribution. But the sum mentioned was the net result of the whole position, and the gross deficiency of those hospitals which had deficiencies was much larger. To achieve the result Major Matthews has in mind it would be necessary for a hospital with a surplus to hand it over for the benefit of the less fortunate institutions. This would be rather hard on the hospital which had a good year after several bad ones, or on the institution faced by a deficit in 1920. Were it granted that the time for State help has arrived the system of its application is not so simple as Major Matthews appears to think.

### PERSONAL SERVICE AND COTTAGE HOSPITALS.

No hospitals have a better and more unobtrusive record of good work than our cottage hospitals, and it is a pleasure to note the loyalty with which they are served. Thus Mr. C. Burlingham has resigned after forty years' work as honorary secretary of Evesham Cottage Hospital, and Mr. J. Bracher has also retired after a similar period, during which he has been honorary treasurer. Both, in fact, have held office since the hospital was started. Resolutions of thanks and appreciation are to be engrossed and signed for presentation to them. Their retirement marks a chapter in the history of the institution, which is now to be known as the Evesham Hospital. Mr. L. C. Cox is the new honorary secretary, but the need for a paid official is admitted.

### LIVERPOOL DENTAL HOSPITAL.

During the war this institution did a great work, granting free treatment to those Territorials in and around Liverpool who had been rejected by the Army because of their defective teeth. Mr. Golotti, the treasurer, raised a fund to pay the expenditure thus incurred. Moreover, the committee arranged with the Admiralty that all naval ratings using the port of Liverpool should receive their dental treatment at the hospital. Since then the hospital has become a centre for training discharged men to be dental mechanics.

### THE BABIES' HOSPITAL AT HARROGATE.

The child-welfare movement, which was begun in Harrogate in 1914, has now extended by the opening of the Harrogate Babies' Hospital. This was originally the convalescent home attached to the war hospitals of the Grand Duchess George of Russia. Mr. J. Shepherd, who is not only the Mayor, but the Chairman of the Health Committee of the Corporation, said that the latter had virtually purchased the premises for £3,000, and was allowing them £900 for the coming year. Babies are already in the hospital and several hundred are upon the welfare roll. Dr. Mair, the medical officer of health, records that the infant mortality rate had fallen since 1900 from 127 to 51 per thousand. This shows the value of welfare work.

### CONSUMPTIVE CHILDREN BEST AT SCHOOL.

The annual report of the Hull School Medical Officer confirms what has been said in a previous report as to the advantage of children suffering from pulmonary tubercle attending school when this can be done without risk to their fellow-pupils. Last year the tuberculosis officer was able to certify as fit to return to school 107 children, much the larger proportion of these showing signs of arrested disease or no signs of disease at all. Many of these children had been absent for months, or even years, on the excuse of requiring as much fresh air as possible. But, as in most cases this would only mean occasional walks or playing in the streets, varied by sitting over the fire in a stuffy room, it is obvious that the child is far better off when attending school in a properly warmed and ventilated class-room, and passing most of its time there in bodily rest. A further consideration is that it is also not losing valuable time from its education, and is not contracting habits of idleness, if nothing worse, which will have a bad effect on its whole future life.

### WHY SANATORIUM PATIENTS RETURN.

Dr. W. Goodchild, medical superintendent of Blencathra Sanatorium, Cumberland, notes a number of readmissions during the past year. He attributes these to the fact that many of these patients were ex-Service men who had not stayed their full time when first treated in consequence of the restlessness created by the war. Other patients were fit for some work if the right kind of work could have been found for them. The difficulty, however, is great, and so long as many occupations are conducted with insufficient ventilation and in avoidable dust, so long will ex-sanatorium patients lose what they have gained, and drift into a life of idleness. On the other hand, patients of ten or fifteen years ago are heard of in full activity. Some went through the war without relapse or even sickness. Dr. Goodchild is considering how to provide graduated work for his patients, and it will be interesting to hear how he can solve the difficulty of providing work which, fit in itself, can be continued after discharge.

### SANATORIUM FOR ESSEX.

Essex County Council, which has been roused by the needs of the County in regard to the treatment of tuberculosis, has now decided to erect its own sanatorium, and has secured as the site the Rectory Farm of fifty-four acres, at a cost of £3,000. The County Council went into the question of the purchase of an existing institution, and also the purchase of a mansion to adapt, but have decided to build its own establishment.

### NEW SECRETARY AT NORTHERN COUNTIES HOSPITAL.

Another hospital with a balance in hand is the Northern Counties Hospital for Consumption and Diseases of the Chest, which succeeded in increasing its balance to £200 during 1919. This must be a gratifying fact to Mr. J. A. Dees, who has retired at this encouraging moment from the secretaryship which he has held since 1901. The new secretary, Mr. Carlyle Gibson, B.Sc., is confronted with the task of rebuilding. A sum of at least £2,000 is required to begin, and of this some £600 is in hand. Support from the workers in various firms and trades has been promised, including the Northumberland miners, engineers, and railwaymen. The hospital is remarkable from the fact that it treats practically no in-patients. It would be interesting to hear from Mr. Gibson what place would be devoted to them in the new building.

### BRADFORD'S MUNICIPAL HOSPITAL.

The Ministry of Health has now agreed to the proposal of the Bradford Guardians to hand over St. Luke's Hospital to the Bradford Corporation. The hospital possesses 1,200 beds, and Dr. Addison has risked the doubt whether the present powers of municipal bodies allow them to provide general hospitals for infectious cases. The maintenance of St. Luke's Hospital will cost the ratepayers between £100,000 and £120,000 a year, half of which will be paid by the Guardians for the treatment of the sick poor. The consulting staff of the hospital will be paid, and the scheme, of which much has been heard, is at last sanctioned.

### A PATIENT JUDGED BY HIS PEERS.

The patient with a grievance is known in every hospital. Occasionally he or she succeeds in making things very unpleasant for the staff. Such a case has been considered by the Pembrokeshire Pensions Committee at Haverfordwest. His complaints were against St. John's Hospital, Tenby, but on hearing the allegations the institution's patients held a meeting on their own initiative and passed a unanimous resolution of confidence in the staff. According to Dr. Knowling, the medical officer, the man had been discontented since his admission to the hospital, and by his agitation had been the means of making others discontented. The committee, after hearing reports upon the treatment, found that the complainant had no ground for his case. In view of the resolution passed by the other patients, the complainant will have had the satisfaction of being judged not only officially, but by his peers.

### COTTAGE HOSPITALS AND THE RED CROSS.

The Board of the Bridgend (Glam.) Cottage Hospital is contemplating the provision of additional wards and an out-patients' department. There are insufficient funds, however, to complete the full scheme, and pending receipt of a portion of the Red Cross surplus, the hospital management is doing the best it can with the money in hand. Application to the Red Cross has been made and a grant is confidently expected.

### WAR MEMORIAL AT A POOR-LAW INFIRMARY.

Southwark Board of Guardians, subject to the sanction of the Ministry of Health, proposes to erect a war memorial at the infirmary. The memorial will record the fact that the infirmary was used for a military hospital during the war, and will commemorate the loss to the Empire of the soldiers who died there. Made of selected Portland stone fixed on concrete foundations, it will be twelve feet high, and will be erected in the grounds in front of the infirmary. The cost is expected to be £285. Is this the first war memorial in a Poor-Law infirmary?

### AN ALMONER AT THE LONDON HOMŒOPATHIC.

The committee of the London Homœopathic Hospital has decided to appoint an almoner to inquire into the circumstances of the patients, so that each may be asked to contribute according to his means. When the appointment has been made the work will no doubt lead also to the assistance of patients after their discharge. It is important that the almoner's office should not be regarded as merely inquisitorial.

### PUSSYFOOT IN FRENCH INDIA.

A very important change has just been introduced by the French Government in their revenue administration in the French settlement in Malabar by (1) reducing the number of taverns for sale of country and foreign liquor; (2) prohibiting the manufacture of country spirit out of toddy, which was a monopoly enjoyed by the French citizens; (3) raising the price of all liquors to the level of those prevailing in British India. Hitherto the French colony in Malabar was a favourite resort by reason of the marvellously cheap prices at which foreign and country liquors were available to the public; and the British Government has been compelled to levy a heavy customs' duty on liquors imported from French to British territory. With the present change in the revenue policy of the French Government the British Government have been enabled to abolish the customs' duties on liquors.

### LONDON TEACHERS' AID FOR THE HOSPITALS.

The London Teachers' Association has assured the County Council that teachers and pupils alike will welcome an opportunity of helping the hospitals and will support an organised appeal. Arrangements have accordingly been made for representative teachers to meet representatives of King Edward's Hospital Fund to discuss how best to organise a general hospital appeal in the Council schools, and to decide upon the distribution of the amount collected.

### NEW COTTAGE HOSPITAL FOR SOUTH WALES.

Ammanford, a colliery town about eighteen miles from Swansea, has decided to convert the Y.M.C.A. buildings started there into a cottage hospital. The Amman Valley Cottage Hospital was originally designed to serve the whole area. Ammanford has now decided to proceed on its own. It is stated that the Y.M.C.A. buildings can be acquired for less than £4,000, and have been approved by Dr. Isaac, Mr. Brook and members of the Swansea Hospital House Committee.

### THIS WEEK'S DRUG MARKET.

Business has not yet recovered from the Easter holidays and is not likely to be active again for a week or so. The general upward tendency of prices is in the meantime in abeyance, and a lower tendency is noticeable in the case of several articles. For instance, Japanese refined camphor is selling at lower prices, and Japanese peppermint oil is also cheaper. Other drugs which are offered at rather lower rates include castor oil, salol, calumba root, clove oil, and orris root. On the other hand, citric acid, tartaric acid, cream of tartar, phenacetin and opium still have an upward price tendency. German bromide of potassium is more freely offered, and the price is lower. Cocaine is in good demand, and the price tends upwards. The scarcity of hexamine continues. The high value of saffron is firmly maintained.

